# Induction, Purification and Characterization of a Novel Manganese Peroxidase from *Irpex lacteus* CD2 and Its Application in the Decolorization of Different Types of Dye

**DOI:** 10.1371/journal.pone.0113282

**Published:** 2014-11-20

**Authors:** Xing Qin, Jie Zhang, Xiaoyu Zhang, Yang Yang

**Affiliations:** College of Life Science and Technology, Huazhong University of Science and Technology, Wuhan, 430074, China; Russian Academy of Sciences, Institute for Biological Instrumentation, Russian Federation

## Abstract

Manganese peroxidase (MnP) is the one of the important ligninolytic enzymes produced by lignin-degrading fungi which has the great application value in the field of environmental biotechnology. Searching for new MnP with stronger tolerance to metal ions and organic solvents is important for the maximization of potential of MnP in the biodegradation of recalcitrant xenobiotics. In this study, it was found that oxalic acid, veratryl alcohol and 2,6-Dimehoxyphenol could stimulate the synthesis of MnP in the white-rot fungus *Irpex lacteus* CD2. A novel manganese peroxidase named as CD2-MnP was purified and characterized from this fungus. CD2-MnP had a strong capability for tolerating different metal ions such as Ca^2+^, Cd^2+^, Co^2+^, Mg^2+^, Ni^2+^ and Zn^2+^ as well as organic solvents such as methanol, ethanol, DMSO, ethylene glycol, isopropyl alcohol, butanediol and glycerin. The different types of dyes including the azo dye (Remazol Brilliant Violet 5R, Direct Red 5B), anthraquinone dye (Remazol Brilliant Blue R), indigo dye (Indigo Carmine) and triphenylmethane dye (Methyl Green) as well as simulated textile wastewater could be efficiently decolorized by CD2-MnP. CD2-MnP also had a strong ability of decolorizing different dyes with the coexistence of metal ions and organic solvents. In summary, CD2-MnP from *Irpex lacteus* CD2 could effectively degrade a broad range of synthetic dyes and exhibit a great potential for environmental biotechnology.

## Introduction

Manganese peroxidase (MnP, EC 1.11.1.13) is the heme-containing glycoprotein which is mainly produced by white-rot fungi such as *Phanerochaete chrysoporium*, *Ceriporiopsis subvervispora*, *Dichomitus squalens*, *Pleurotus ostreatus*, *Pleurotus pulmonarius*, *Pleurotus eryngii*. The MnP, which is the important component of extracellular ligninolytic enzymes of lignin-degrading fungi, can catalyze the H_2_O_2_-dependent oxidation of Mn^2+^ into Mn^3+^, and then chelates of Mn^3+^ with fungal organic acid cause one-electron oxidation of various compounds (A schematic representation of the enzyme reaction was shown in Fig.S1). MnP has the strong ability of oxidizing and depolymerizing natural and synthetic lignins [1]–[3]. Besides the use in the conversion of lignin and lignocelluloses [2], MnP has great application potential in the field of environmental biotechnology and degradation of some recalcitrant organopollutants that are very harmful to human health, such as polycyclic aromatic hydrocarbons [4], [5], chlorophenols [6], industrial dyes [7]–[10] and nitroaromatic compounds [11]. The great value of MnP in the application in bioremediation results in more and more attention to this enzyme.

The unique degradative ability of MnP makes this enzyme valuable for various biotechnological applications. Thus, in recent years, some MnPs have been purified and characterized from different fungal strains such as *Agrocybe praecox* [12], *Dichomitus squalens* [8], *Irpex lacteus* [4], [13], *Stereum ostrea* [14], *Phanerochaete chrysosporium* [15], *Lentinula edodes* [16], *Schizophyllum* [7]. The enzymatic properties of these purified MnPs from different sources have been studied. Previous research has demonstrated that some azo and anthraquinone dyes, polycyclic aromatic hydrocarbons (phenanthrene, anthracene, fluoranthene, and pyrene), 2,4,6-trinitrotoluene can be efficiently degraded by the purified MnPs from *Dichomitus squalens* [8], *Stereum ostrea* [14], *Irpex lacteus* [4] and *Phlebia radiate* [11]. The ability of nanoclay-immobilized MnP from *Anthracophyllum discolor* to degrade polycyclic aromatic hydrocarbons [5] and the capability of sol–gel matrix immobilized MnP from *Ganoderma lucidum* for decolorization of different dye effluents have also been evaluated [17].

Although there have been some reports about the properties of purified MnPs and their application in the enzymatic degradation of environmental pollutants as described above, some other factors have to be considered for the more efficient application of MnP in the area of biodegradation. For example, the dye effluents discharged by textile industry usually contain high level of different metal ions and organic solvents. Thus, the ability of MnP to tolerate different metal ions or organic solvents is very important for the efficient application of this enzyme in the treatment of wastewater. However, to our knowledge, few studies have been performed to evaluate the capability of purified MnP for tolerating different metal ions and organic solvents. Most previous research mainly focused on the enzymatic and kinetic properties of MnP purified from different fungi [12], [13], [15], [16]. Therefore, searching for new MnP with stronger tolerance to metal ions and organic solvents is important for the maximization of potential of MnP in the biodegradation of recalcitrant xenobiotics.

The white-rot fungi *Irpex lacteus* has been shown to demonstrate a significant potential for the various biotechnological applications such as bioremediation of organopollutants in water and soil environments, degradation of different lignocellulosic substrates yielding higher sugar recoveries compared to other fungal treatments. The great application values of *Irpex lacteus* are attributed to the extracellular peroxidase including manganese peroxidase, versatile peroxidase and dye-decolorizing peroxidase [18], [19]. In the previous research of our laboratory, a new white-rot fungi strain *Irpex lacteus* CD2 has been isolated and characterized from the Shennongjia Nature Reserve of Hubei Province in China [20]–[22]. The effect and mechanism of biopretreatment of cornstalks by *Irpex lacteus* CD2 have been intensively studied in our laboratory [20]–[22]. For the purpose of better use of this fungus and its manganese peroxidase in the field of environmental biotechnology, in this work, the properties of the purified manganese peroxidase (named as CD2-MnP) from *Irpex lacteus* CD2 and its ability to decolorize different types of dyes and simulated textile wastewater were investigated. We also focused on the evaluation of the capability of this MnP for tolerating different metal ions and organic solvents. In addition, the capability of CD2-MnP to decolorize different dyes with the coexistence of metal ions and organic solvents was further assessed.

## Materials and Methods

### Dyes and Chemicals

The different types of dyes used in this study were purchased from Aldrich-Sigma (USA). All of other chemicals were of analytical grade and obtained from Sinopharm Chemical Reagent Company (China).

### Strains and culture conditions

The white rot fungus *Irpex lacteus* CD2 was characterized in the previous work of our laboratory [20]–[22]. It was maintained at 4°C on potato dextrose agar (PDA) slant. The inoculum was grown in potato dextrose broth (PDB) medium for 7 days at 28°C, then cultures were transferred into the basal liquid medium as a 10% (v/v) inoculum and incubated at 28°C in a shaking incubator (150 rpm). The basal liquid medium contained (g/L): Glucose 20 g, Yeast extract 2.5 g, KH_2_PO_4_ 1 g, Na_2_HPO_4_ 0.05 g, MgSO_4_·7H_2_O 0.5 g, CaCl_2_ 0.01 g, FeSO_4_·7H_2_O 0.01 g, MnSO_4_·4H_2_O 0.001 g, ZnSO_4_·7H_2_O 0.001 g, CuSO_4_·5H_2_O 0.002 g [23].

### Measurement of MnP activity and protein contents

Manganese peroxidase activity was measured by monitoring the formation of Mn^3+^-malonate complexes at 270 nm as described previously [24]. The assay mixture contained 1 ml of 4 mM MnSO_4_, 1 mL of 20 mM malonate buffer (pH 5.0), 0.5 mL of 0.4 mM H_2_O_2_ and 0.1 mL of enzyme solution. One unit of enzyme activity was defined as the amount of enzyme that oxidized 1 µmol of Mn^2+^ per min at 30°C. Protein contents were determined by the method of Bradford using BSA as the standard.

### Induction of manganese peroxidase produced by *Irpex lacteus* CD2

The fungus was grown at 28°C with shaking at 150 rpm for 5 days. Then the following inducers including oxalic acid, veratryl alcohol, 2,6-dimethoxyphenol were respectively added into the actively growing 5-day-old cultures of *Irpex lacteus* CD2 at the final concentration of 100 mg/L. After adding the inducers, the fungal cultures were then grown at 28°C with shaking at 150 rpm continuously. Samples were withdrawn every day, centrifuged, and the clear supernatant was used for measuring the extracellular MnP activity.

### Purification of manganese peroxidase named as CD2-MnP from *Irpex lacteus* CD2

The liquid cultures of *Irpex lacteus* CD2 at the peak of MnP activity were collected and centrifuged at 5000 g for 20 min. Then the culture supernatant was concentrated by 80% ammonium sulfate at 4°C. The sodium acetate buffer (20 mM, pH 4.8) was used to dissolve the pellets. The enzymatic crude extract was dialyzed to remove ammonium sulfate and then applied to a DEAE Sepharose Fast Flow anion exchange column (GE) equilibrated with sodium acetate buffer (20 mM, pH 4.8). The MnP was eluted with a linear gradient of 0–1 M NaCl in the same buffer at a flow rate of 1 ml/min. The proteins in the eluted fractions was detected by recording the absorbance at 280 nm continuously. Active fractions containing MnP activity were pooled, desalted, filter-sterilized, and stored at 4°C. The purified MnP was verified by SDS-PAGE using 10% polyacrylamide gel. The molecular mass of the purified MnP was estimated by protein ladder molecular weight markers.

### Characterization of purified CD2-MnP

Kinetic studies were performed in 20 mM malonate buffer (pH 4.5) at 30°C using 5–150 µM Mn^2+^ (in the presence of 0.08 mM H2O2), 4–80 µM hydrogen peroxide (in the presence of 1.6 mM Mn^2+^) as substrates. The Lineweaver–Burk plot method was used to determine Km and Vmax of the purified CD2-MnP.

The UV-visible spectrum of purified CD2-MnP, in 20 mM malonate buffer (pH 5.0), was measured in the range from 300 nm to 800 nm (UV-1600PC Spectrophtometer, Apada).

The effect of temperature on MnP activity was measured in 20 mM malonate buffer (pH 4.5) at 20–80°C. The effect of pH on MnP activity was determined in 20 mM malonate buffer within a pH range of 3.0–7.0 at 30°C. The maximum activity of MnP was set as 100%.

To evaluate the thermal stability, the purified MnP was incubated at 40–70°C for 5 h. To evaluate the pH stability, the purified MnP was incubated in different pH (3–6) for 6 h and 24 h. Then the residual MnP activity was calculated based on the original activity before incubation. The initial activity of MnP was set as 100%.

Al^3+^, Ca^2+^, Cd^2+^, Co^2+^, Mg^2+^, Ni^2+^ and Zn^2+^ (at concentration of 0.4 mM, 2 mM, 4 mM and 40 mM) were used to study the effect of metal ions on the activity of purified MnP. The residual activity was calculated based on the control without adding any metal compound (set as 100%).

Methanol, ethanol, DMSO, ethylene glycol, isopropyl alcohol, butanediol, glycerin and acetonitrile (at concentration of 10%, 20% and 30%) were used to study the effect of organic solvents on the activity of purified MnP. The residual activity was calculated based on the control without adding any organic solvent (set as 100%).

To evaluate the effect of different metal ions on the stability of purified MnP, the purified MnP was incubated with different concentrations of metal ions (0.4 mM and 4 mM) at 25°C for 12 h and 24 h respectively. Then the MnP activity was measured. The residual activity was calculated based on the control without adding any metal compound (set as 100%).

To evaluate the effect of different organic solvents on the stability of purified MnP, the purified MnP was incubated with different concentrations of organic solvents (10% and 30%) at 25°C for 12 h and 24 h respectively. Then the MnP activity was measured. The residual activity was calculated based on the control without adding any organic solvent (set as 100%).

All of above experiments were performed in triplicate.

### Decolorization of different types of dyes by purified CD2-MnP

To evaluate the dye decolorization capability of the purified MnP, the purified CD2-MnP was used to decolorize four types of synthetic dyes including azo dye Remazol Brilliant Violet 5R and Direct Red 5B, triphenylmethane dye Methyl Green, anthraquinone dye Remazal Brilliant Blue R and indigo dye Indigo Carmine. The reaction mixture in a total volume 1 ml contained (final concentration): malonate buffer (20 mM, pH 4.5), Mn^2+^ (1.6 mM), H_2_O_2_ (0.08 mM), purified CD2-MnP (0.25 U/ml) and dye (50 mg/L). Decolorization was monitored by measuring the absorbance of the reaction mixture at 556 nm for Remazol Brilliant Violet 5R, 510 nm for Direct Red 5B, 640 nm for Methyl Green, 600 nm for Remazal Brilliant Blue R, 610 nm for Indigo Carmine. The decolorization of dye, expressed as dye decolorization (%), was calculated as the following formula: decolorization (%) = [(Ai-At)/Ai]*100, where Ai is the initial absorbance of the dye and At is the absorbance of the dye at time t [25].

To evaluate the effect of different metal ions on the decolorization of dyes by purified CD2-MnP, the reaction mixture in a total volume of 1 ml contained (final concentration): malonate buffer (20 mM, pH 4.5), Mn^2+^ (1.6 mM), H_2_O_2_ (0.08 mM), purified CD2-MnP (0.25 U/ml), dye (50 mg/L), Ca^2+^, Co^2+^, Mg^2+^, Zn^2+^ (4 mM). Then decolorization was monitored and calculated by the method described above.

To evaluate the effect of different organic solvents on the decolorization of dyes by purified CD2-MnP, the reaction mixture in a total volume of 1 ml contained (final concentration): malonate buffer (20 mM, pH 4.5), Mn^2+^ (1.6 mM), H_2_O_2_ (0.08 mM), purified CD2-MnP (0.25 U/ml), dye (50 mg/L), methanol, DMSO, ethylene glycol, glycerin (20%). Then decolorization was monitored and calculated by the method described above. All of the decolorization experiments were performed in triplicate.

### Decolorization of simulated textile wastewater by purified CD2-MnP

Simulated textile wastewater containing Remazol Brilliant Violet 5R, Direct Red 5B, Remazal Brilliant Blue R and Indigo Carmine was prepared as described in reference [26]. The stimulated textile wastewater containing different dyes was prepared as follows: 0.5 g L^−1^ dye, 30 g L^−1^ NaCl, 5 g L^−1^ Na_2_CO_3_ and 1.5 mL L^−1^ 35% w/v NaOH, the pH was adjusted to 4.5. The reaction mixture in a total volume of 1 ml contained (final concentration): malonate buffer (20 mM, pH 4.5), Mn^2+^ (1.6 mM), H_2_O_2_ (0.08 mM), purified CD2-MnP (0.5 U/ml), simulated textile wastewater (10%, 30%, 50%). Then decolorization was monitored and calculated by the method described above. The decolorization of simulated textile waste was measured by monitoring the decrease in maximum absorbance of each wastewater in a UV-vis spectrophotometer.

### Statistical analysis

To evaluate the effects of metal ions and organic solvents on MnP activity and decolorization of dyes, the ANOVA, the analysis of variance, was performed using the software SPSS (*significant difference, p-value<0.05; **highly significant difference, p-value<0.01).

## Results

### Induction and purification of manganese peroxidase from *Irpex lacteus* CD2

It is known that the aromatic compounds play an important role in the regulation of the ligninolytic enzymes synthesis [27]. Besides, organic acids are oxidised by MnP to produce extracellular hydrogen peroxide, which can stimulate the manganese peroxidase gene transcription [28]. To enhance the production of extracellular MnP by *Irpex lacteus* CD2, the effect of different lignin monomer analogs and organic acids on the activity of extracellular MnP was studied. Time course of MnP activity after addition of various inducers was shown in the Fig.S2. As shown in Fig.S2, the maximum activity of MnP occurred at the 7th day after addition of inducers. Oxalic acid and veratryl alcohol could significantly enhance the synthesis of extracellular MnP produced by *Irpex lacteus* CD2. The highest MnP activity was observed in the cultures supplemented with oxalic acid (640.7 U/L), veratryl alcohol (549.0 U/L) and 2,6-dimethoxyphenol (273.5 U/L) (Fig.S2).

**Figure 1:**
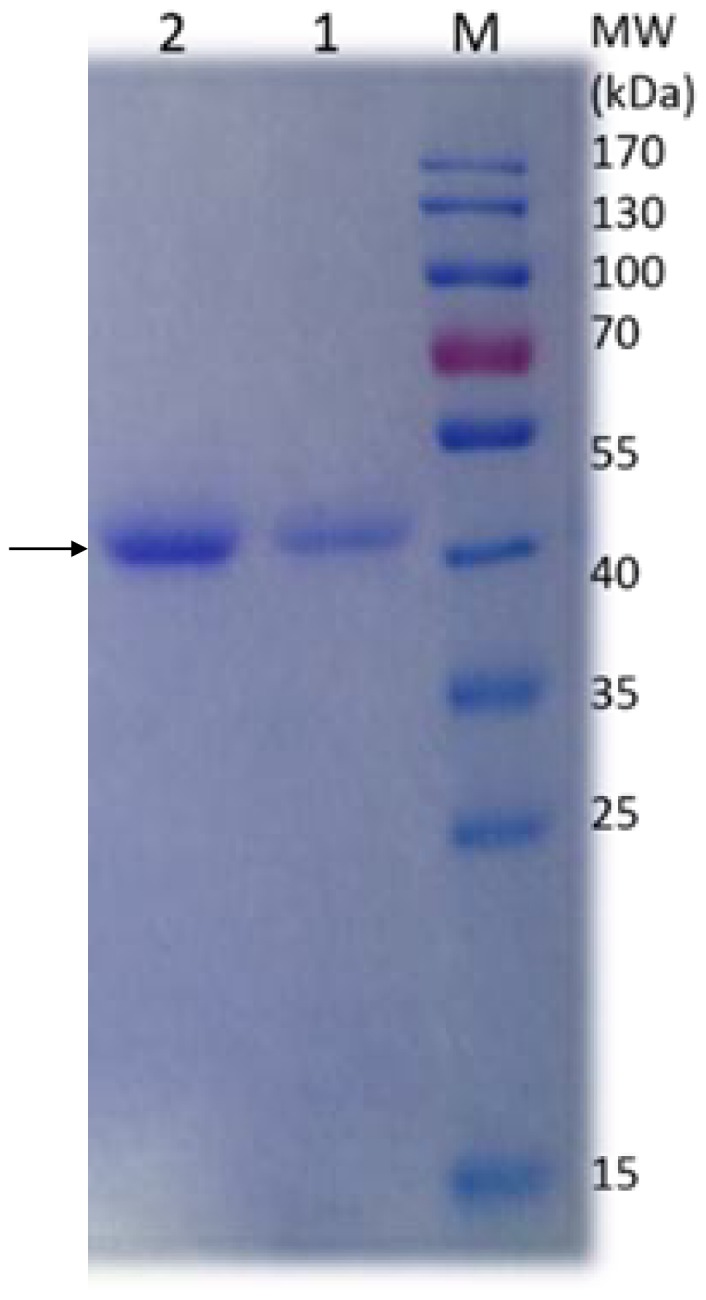
SDS-PAGE analysis of the purified MnP from *Irpex lacteus* CD2. lane M: molecular mass marker; lane 1 and lane 2: purified MnP.

The MnP secreted by *Irpex lacteus* CD2 was then purified as described in Table 1. This MnP named as CD2-MnP was purified over 29.3-fold with a terminal specific activity of 24.9 U/mg protein. The result of SDS-PAGE suggested that this enzyme was purified to homogeneity. The molecular mass of CD2-MnP was about 42 kDa as determined by SDS-PAGE (Fig. 1).

**Table 1:** Purification of manganese peroxidase from *Irpex lacteus* CD2.

Purification step	Total Protein (mg)	Total activity(U)	Specific activity(U/mg)	Yield (%)	Fold
Culture filtrate	278.08	235.84	0.85	100.00	1.00
Concentration	32.84	224.64	6.84	95.25	8.07
DEAE-Sepharose,pH 4.8	2.79	69.50	24.91	29.47	29.37

### Kinetic studies on the purified CD2-MnP

The kinetic parameters of CD2-MnP with respect to hydrogen peroxide and Mn^2+^ were determined. The Km values of CD2-MnP were 20.72 µM for H_2_O_2_ and 49.41 µM for Mn^2+^.

### UV-visible spectrum of the purified CD2-MnP

**Figure 2:**
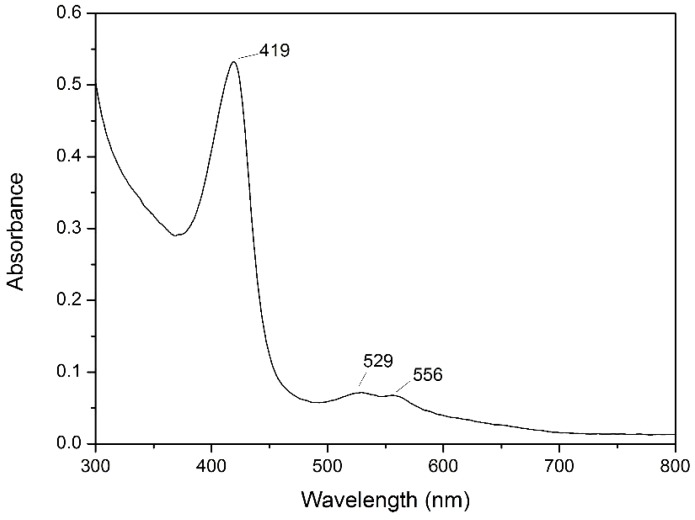
UV-visible spectrum of the purified CD2-MnP.

Like heme peroxidase including horseradish peroxidase and lignin peroxidase, the catalytic cycle of MnP included the native ferric enzyme and the reactive intermediate compound I, II [2]. The identification of oxidized states of MnP compounds I, II was reported by different absorption maxima [29]. As shown in Fig.2, the absorption spectrum of purified CD2-MnP from *Irpex lacteus* CD2 showed maxima at 419 nm, 529 nm and 556 nm, which suggested that CD2-MnP was a heme protein with iron protoporphyrin IX as compound II.

### Effect of pH on the MnP activity and stability of CD2-MnP

**Figure 3:**
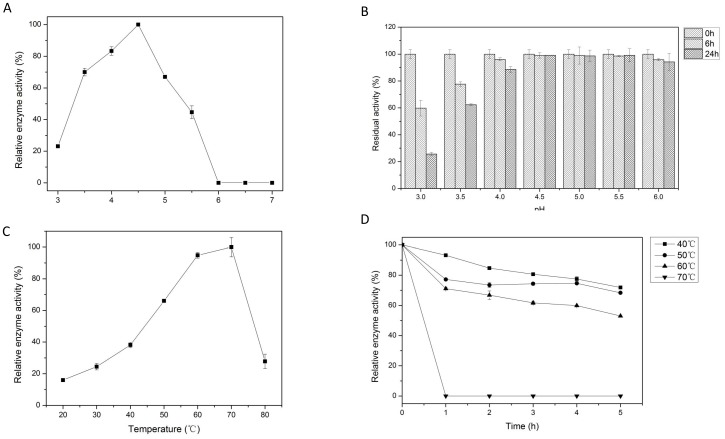
Effect of pH and temperature on the activity and stability of purified CD2-MnP from *Irpex lacteus* CD2. **A**: Effect of pH on MnP activity. The activity of 100% was that which was measured at the optimal pH. **B**: Effect of pH on the stability of CD2-MnP. The initial MnP activity before incubation was set as 100%. **C**: Effect of temperature on MnP activity. The activity of 100% was that which was measured at the optimal temperature. **D**: Effect of temperature on the stability of CD2-MnP. The initial MnP activity before incubation was set as 100%.

The optimal pH for CD2-MnP was 4.5. CD2-MnP was completely inactive when the pH was above 6.0 (Fig.3A). As shown in Fig.3B, CD2-MnP exhibited high stability in pH ranging from 3.5 to 6.0. The residual MnP activity of CD2-MnP after 24 h incubation at pH 3.5, 4, 4.5, 5, 5.5, 6 was 62.4%, 88.7%, 99.1%, 98.7%, 99.2%, 94.2% of the original activity before incubation, respectively.

### Effect of temperature on the MnP activity and stability of CD2-MnP

The optimal temperature of CD2-MnP was determined to be 70°C (Fig.3C). As shown in Fig.3D, CD2-MnP could respectively retain 72.0%, 68.4% and 53.1% of MnP activity after 5 h incubation at 40°C, 50°C and 60°C. When the temperature increased to above 70°C, the thermostability of CD2-MnP significantly decreased.

### Effect of different metal ions on the MnP activity and stability of CD2-MnP

**Figure 4:**
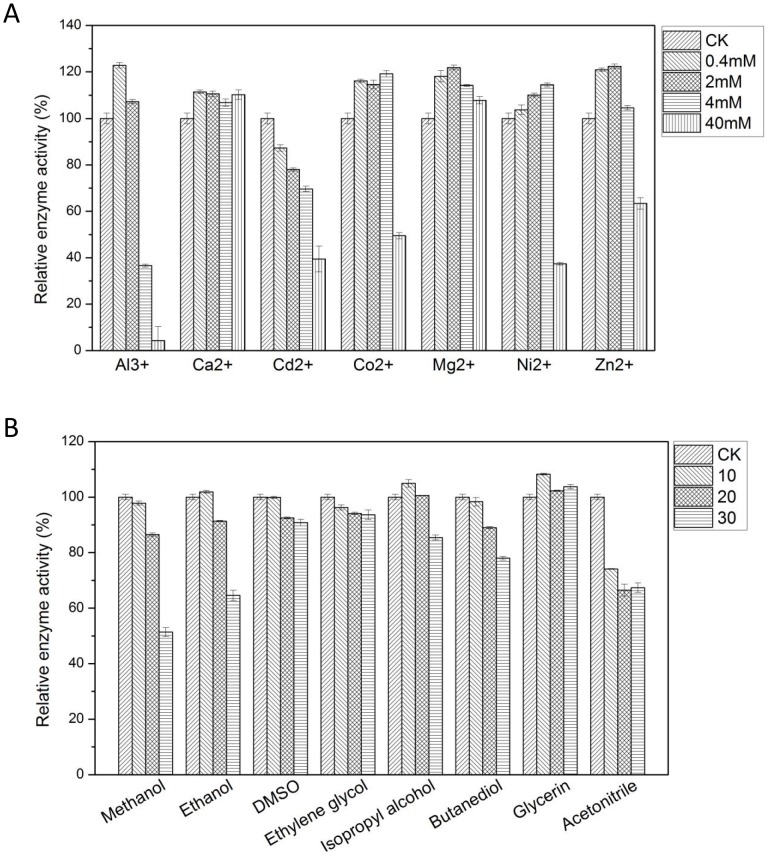
Effect of metal ions and organic solvents on the activity of purified CD2-MnP. **A**: The effect of different metal ions on MnP activity. The MnP activity of the control without adding any metal compound was set as 100%. **B**: The effect of different organic solvents on MnP activity. The MnP activity of the control without adding any organic solvent was set as 100%.

As shown in Fig.4A, low concentrations of metal ions such as Ca^2+^, Cd^2+^, Co^2+^, Mg^2+^, Ni^2+^ and Zn^2+^ had little effect on the MnP activity of CD2-MnP. When the concentration of Ca^2+^, Cd^2+^, Co^2+^, Mg^2+^, Ni^2+^ and Zn^2+^ was 4 mM, the MnP activity of CD2-MnP was 106.8%, 70.0%, 119.1%, 114.1%, 110.0%, 104.5% of the control without adding any metal compound (Fig.4A). But higher concentrations (40 mM) for all metal ions (other than Ca^2+^ and Mg^2+^) resulted in the reduced MnP activity (Fig.4A). It suggested that higher concentrations of metal ions such as Cd^2+^, Co^2+^, Ni^2+^ and Zn^2+^ had an inhibitory effect on the MnP activity of CD2-MnP.

The relative MnP activities of CD2-MnP at different metal ions (final concentration: 40 mM) were compared. As shown in Fig.S3, compared with the relative activity of CD2-MnP at 40 mM Ca^2+^ and Mg^2+^ (111.5% and 107.1%), the relative activity of CD2-MnP at 40 mM Al^3+^, Cd^2+^, Co^2+^, Ni^2+^ was much lower (8.6%, 39.5%, 49.5%, 37.4%). The MnP activities of CD2-MnP at 40 mM Al^3+^, Cd^2+^, Co^2+^, Ni^2+^ were significantly lower than that of CD2-MnP at 40 mM Ca^2+^ and Mg^2+^ (p-value<0.01) (Fig.S3-A,B). Thus the data obtained by the statistical analyses suggested that CD2-MnP showed stronger tolerance to Ca^2+^ and Mg^2+^ compared to other metal ions.

As shown in Fig.S3, the relative MnP activity of control (without adding any metal compound) was also significantly lower than that of CD2-MnP at 40 mM Ca^2+^ and Mg^2+^ (p-value<0.01) (Fig.S3-A,B). It suggested that higher concentration of Ca^2+^ and Mg^2+^ (40 mM) had no inhibitory effect on the MnP activity of CD2-MnP. In contrast, higher concentration of Ca^2+^ and Mg^2+^ could enhance the MnP activity of CD2-MnP.

As shown in Table 2, CD2-MnP exhibited good stability in different metal ions.

**Table 2:** Effect of metal ions on the stability of purified CD2-MnP from *Irpex lacteus* CD2.

Chemicals	Concentration (mM)	Relative enzyme activity(%)	
		12 h	24 h
None		100%	100%
CoCl_2_	0.40	101.48	96.96
	4.00	102.92	98.98
CaCl_2_	0.40	101.90	101.29
	4.00	101.81	102.04
CdCl2	0.40	97.05	94.16
	4.00	107.24	100.57
MgCl_2_	0.40	104.00	102.22
	4.00	106.04	105.43
AlCl_3_	0.40	103.27	101.75
	4.00	107.34	108.14
NiCl_2_	0.40	99.26	100.23
	4.00	96.78	96.83
LiCl	0.40	107.80	99.30
	4.00	103.72	95.14
ZnCl_2_	0.40	105.80	110.78
	4.00	106.44	103.17
FeCl_3_	0.40	102.63	98.60
	4.00	97.78	90.00

The MnP activity of control without adding any metal compound was set as 100%.

CD2-MnP was stable in all of the metal ions tested here when the concentration was 0.4 and 4 mM. It remained about 95% or even higher residual activity after incubation with different metal ions for 24 h (Table 2).

### Effect of different organic solvents on the MnP activity and stability of CD2-MnP

As shown in Fig.4B, when the concentration of organic solvents was 10% and 20%, different organic solvents such as methanol, ethanol, DMSO, ethylene glycol, isopropyl alcohol, butanediol, glycerin had little effect on the MnP activity of CD2-MnP. When the concentration of methanol, ethanol, DMSO, ethylene glycol, isopropyl alcohol, butanediol, glycerin was 20%, CD2-MnP could retain 86.5%, 91.4%, 92.5%, 94.1%, 100.6%, 90.0%, 102.2% residual activity relative to control, respectively (Fig.4B). Acetonitrile had an slight inhibitory effect on the MnP activity of CD2-MnP. CD2-MnP especially exhibited strong tolerance to glycerin, DMSO, ethylene glycol and isopropyl alcohol. When the concentration was increased to 30%, the activity of CD2-MnP could still retain 103.7% (glycerin), 91.0% (DMSO), 93.6% (ethylene glycol) and 86.0% (isopropyl alcohol) relative to the control without adding any organic solvent (Fig.4B).

The stability of CD2-MnP in organic solvents was also studied and showed in Table 3. CD2-MnP remained stable in all of the organic solvents tested here at the concentration of 10%. After 24 h incubation with methanol, ethanol, DMSO, ethylene glycol, isopropyl alcohol, butanediol, glycerin and acetonitrile (final concentration: 10%), the residual activity of CD2-MnP retained 82.6%, 91.7%, 90.5%, 72.5%, 77.2%, 119.3%, 85.4% and 80.0%, respectively. When the concentration of organic solvents was increased to 30%, the stability of CD2-MnP decreased. But CD2-MnP still remained relative stable in ethanol, DMSO, butanediol, and glycerin. The residual activity of CD2-MnP still retained over 80% after 24 h incubation with 30% of ethanol, DMSO, butanediol, and glycerin (Table 3).

**Table 3:** Effect of organic solvents on the stability of purified CD2-MnP from *Irpex lacteus* CD2.

Organic solvents	Concentration	Relative enzyme activity(%)	
		12 h	24 h
None		100%	100%
Methanol	10%	91.90	82.62
	30%	90.48	69.11
Ethanol	10%	102.97	91.77
	30%	98.94	87.01
DMSO	10%	94.87	90.54
	30%	94.04	80.68
Ethylene glycol	10%	85.28	72.54
	30%	83.00	58.42
Isopropyl alcohol	10%	85.34	77.24
	30%	29.21	10.24
Butanediol	10%	134.74	119.33
	30%	129.13	84.42
Glycerin	10%	92.83	85.41
	30%	84.96	80.50
Acetonitrile	10%	95.24	80.02
	30%	26.72	22.35

The MnP activity of control without adding any organic solvent was set as 100%.

In summary, above results suggested that CD2-MnP had a strong ability to tolerate many organic solvents and metal ions. From the viewpoint of practical applications, the strong resistance to different metal ions and organic solvents was a very valuable advantage of CD2-MnP.

### Decolorization of different dyes by the purified CD2-MnP

**Figure 5:**
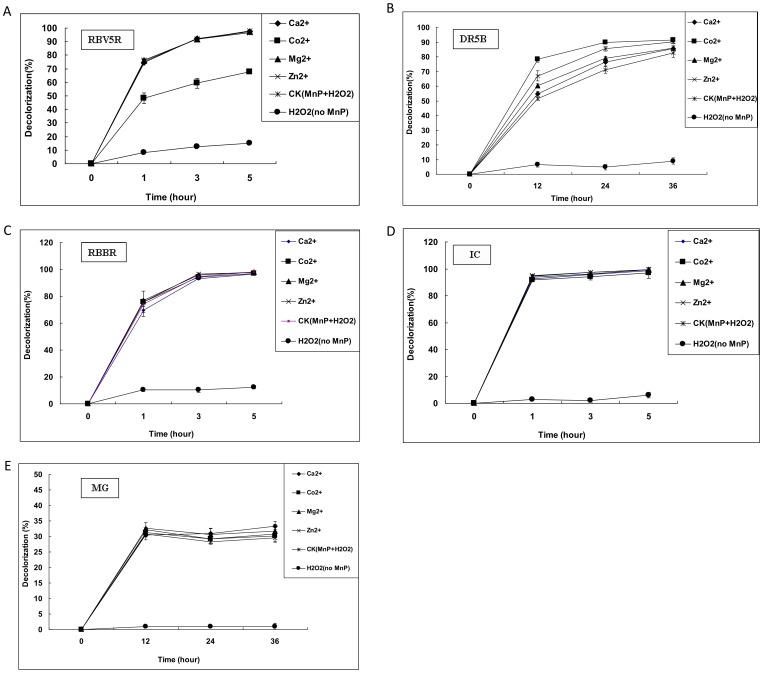
Decolorization of different types of dyes by the purified CD2-MnP with the coexistence of metal ions. The reaction mixture in a total volume 1 ml contained (final concentration): malonate buffer (20 mM, pH 4.5), Mn^2+^ (1.6 mM), H_2_O_2_ (0.08 mM), purified CD2-MnP (0.25 U/ml), dye (50 mg/L) and Ca^2+^, Co^2+^, Mg^2+^, Zn^2+^ (4 mM). **CK (MnP+H_2_O_2_)** was the control without addition of any metal compound except Mn^2+^. **H_2_O_2_ (no MnP)** was the negative control without addition of purified CD2-MnP. (**A**): Decolorization of RBV5R; (**B**): Decolorization of DR5B; (**C**): Decolorization of RBBR; (**D**): Decolorization of IC; (**E**): Decolorization of MG. **RBV5R**: Remazol Brilliant Violet 5R, **DR5B**: Direct Red 5B, **RBBR**: Remazol Brilliant Blue R, **IC**: Indigo Carmine, **MG**: Methyl Green. The negative control (H_2_O_2_ was added into the decolorization mixture in the absence of purified CD2-MnP) showed no significant decolorization of different dyes.

The different types of dyes including the azo dye (Remazol Brilliant Violet 5R, Direct Red 5B), triphenylmethane dye (Methyl Green), anthraquinone dye (Remazol Brilliant Blue R) and indigo dye (Indigo Carmine) were used to evaluate the dye decolorization capability of CD2-MnP. As shown in Fig.5, CD2-MnP showed a strong decolorization capability for a broad range of dyes. Remazol Brilliant Violet 5R, Remazol Brilliant Blue R and Indigo Carmine (50 mg/l) could be respectively decolorized up to 92.8%, 87.1% and 91.5% by the purified CD2-MnP within 5 h (Fig.5A,C,D). Direct Red 5B and Methyl Green (50 mg/l) could be respectively decolorized up to 82.4% and 32.0% by the purified CD2-MnP within 36 h (Fig.5B,E).

CD2-MnP especially exhibited stronger ability to decolorize Indigo Carmine, Remazol Brilliant Violet 5R and Remazol Brilliant Blue R. As shown in Fig.5A,C,D, Indigo Carmine, Remazol Brilliant Violet 5R and Remazol Brilliant Blue R could be decolorized up to 90.5%, 75.3% and 72.1% by CD2-MnP within only 1 h. Compared with the monoazo dye (Remazol Brilliant Violet 5R), anthraquinone dye (Remazol Brilliant Blue R) and indigo dye (Indigo Carmine), it was found that the disazo dye (Direct Red 5B) and triphenylmethane dye (Methyl Green) was harder to be decolorized by CD2-MnP. There was limited decolorization in the negative control (H_2_O_2_ was added into the decolorization mixture in the absence of purified CD2-MnP). For example, as shown in Fig.5A,C,D, Remazol Brilliant Violet 5R, Remazol Brilliant Blue R and Indigo Carmine were respectively decolorized up to 12.0%, 10.3% and 6.1% in the negative control within 5 h.

### Decolorization of different dyes by the purified CD2-MnP with the coexistence of metal ions

In order to investigate the capability of CD2-MnP for decolorizing different dyes at the conditions of high concentrations of metal ions and organic solvents, CD2-MnP was further used to decolorize different dyes with the coexistence of metal ions or organic solvents.

The decolorization of different dyes in the presence of metal ions were tested. As shown in Fig.5A and Fig.S4, the maximum decolorization of RBV5R with the coexistence of Ca^2+^ (within 5 h) was 97.6%, which was higher than that of control without adding any metal compounds (96.6%) (significant difference, p-value<0.05). The maximum decolorization of RBV5R with the coexistence of Zn^2+^ (within 5 h) was 98.0%, which was higher than that of control (96.6%) (highly significant difference, p-value<0.01). Above results suggested that Ca^2+^ and Zn^2+^ had a promotion effect on the ability of CD2-MnP to decolorize RBV5R. As shown in Fig.5A and Fig.S4, the maximum decolorization of RBV5R when Mg^2+^ was present or absent were very similar (p-value>0.05). It suggested that Mg^2+^ had no inhibitory effect on the ability of CD2-MnP to decolorize RBV5R. As shown in Fig.5A and Fig.S4, the maximum decolorization of RBV5R with the coexistence of Co^2+^ (within 5 h) was 67.7%, which was lower than that of control (96.6%) (highly significant difference, p-value<0.01). It suggested that Co^2+^ had an inhibitory effect on the ability of CD2-MnP to decolorize RBV5R.

As shown in Fig.5B and Fig.S4, the maximum decolorization of DR5B with the coexistence of Co^2+^ (within 36 h) was 91.4%, which was higher than that of control without adding any metal compounds (82.6%) (highly significant difference, p-value<0.01). The maximum decolorization of DR5B with the coexistence of Zn^2+^ (within 36 h) was 90.0%, which was higher than that of control (82.6%) (significant difference, p-value<0.05). Above results suggested that Co^2+^ and Zn^2+^ had a promotion effect on the ability of CD2-MnP to decolorize DR5B. As shown in Fig.5B and Fig.S4, DR5B with the coexistence of Ca^2+^ and Mg^2+^ could be decolorized up to 85.5% and 85.8% within 36 h, respectively. Compared with the control (82.6%), the decolorization percentages were not significantly different (p-value>0.05). Thus Ca^2+^ and Mg^2+^ had no inhibitory effect on the ability of CD2-MnP to decolorize DR5B.

As shown in Fig.5C,D,E and Fig.S4, compared with the control, decolorization of Methyl Green, Remazol Brilliant Blue R and Indigo Carmine with the coexistence of different metal ions were not significantly different (p-value>0.05). Thus the data obtained by the statistical analyses demonstrated that different metal ions such as Ca^2+^, Co^2+^, Mg^2+^ and Zn^2+^ had no inhibitory effect on the capacity of CD2-MnP for decolorizing Methyl Green, Remazol Brilliant Blue R and Indigo Carmine.

### Decolorization of different dyes by the purified CD2-MnP with the coexistence of organic solvents

**Figure 6:**
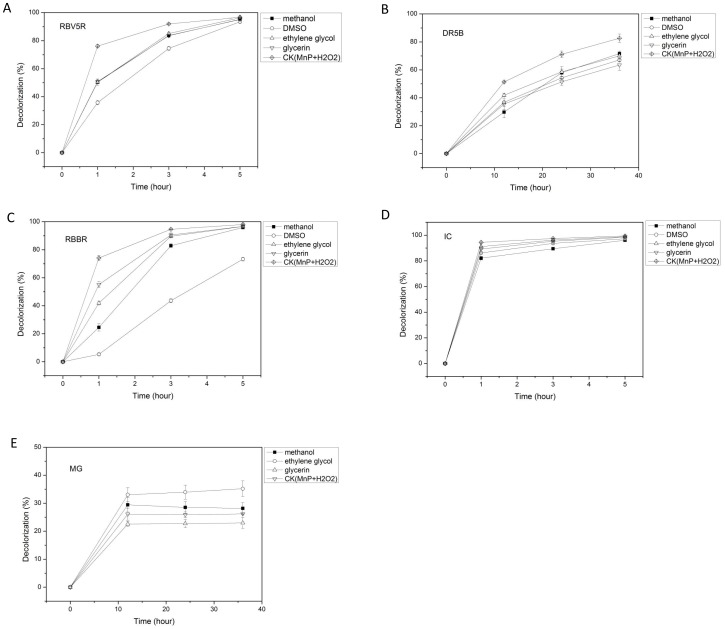
Decolorization of different types of dyes by the purified CD2-MnP with the coexistence of organic solvents. The reaction mixture in a total volume 1 ml contained (final concentration): malonate buffer (20 mM, pH 4.5), Mn^2+^ (1.6 mM), H_2_O_2_ (0.08 mM), purified CD2-MnP (0.25 U/ml), dye (50 mg/L) and methanol, DMSO, ethylene glycol, glycerin (20%). **CK (MnP+H_2_O_2_)** was the control without addition of any organic solvent. (**A**): Decolorization of RBV5R; (**B**): Decolorization of DR5B; (**C**): Decolorization of RBBR; (**D**): Decolorization of IC. (**E**): Decolorization of MG. **RBV5R**: Remazol Brilliant Violet 5R, **DR5B**: Direct Red 5B, **RBBR**: Remazol Brilliant Blue R, **IC**: Indigo Carmine, **MG**: Methyl Green.

The decolorization of different dyes in the presence of organic solvents were also performed. As shown in Fig.6A and Fig.S5, the maximum decolorization of RBV5R with the coexistence of methanol, DMSO, ethylene glycol and glycerin (within 5 h) was 96.0%, 94.0%, 96.4% and 96.0%, which were not significantly different from the control in the absence of any organic solvent (96.6%) (p-value>0.05). It suggested that these organic solvents had no inhibitory effect on the ability of CD2-MnP to decolorize RBV5R.

As shown in Fig.6B and Fig.S5, the maximum decolorization of Direct Red 5B with the coexistence of methanol, DMSO, ethylene glycol and glycerin (within 36 h) were 71.6%, 67.1%, 70.0%, 63.5%, which were much lower than that of control (82.6%) (highly significant difference, p-value<0.01). It suggested that the tested organic solvents had an inhibitory effect on the ability of CD2-MnP to decolorize Direct Red 5B.

As shown in Fig.6C and Fig.S5, when methanol, ethylene glycol and glycerin were present, the maximum decolorization of RBBR within 5 h were 96.0%, 96.7% and 97.0% respectively, which were not significantly different from the control (98.0%) (p-value>0.05). It suggested that methanol, ethylene glycol and glycerin had no inhibitory effect on the ability of CD2-MnP to decolorize RBBR. But the maximum decolorization of RBBR with the coexistence of DMSO (within 5 h) was only 73.2%, which was much lower than that of control (98.0%) (highly significant difference, p-value<0.01). It suggested that the decolorization of RBBR by CD2-MnP was significantly inhibited by DMSO.

As shown in Fig.6D (Indigo Carmine), Fig.6E (Methyl Green) and Fig.S5, the decolorization percentages were not significantly different (between when the organic solvents were present and when they were absent) (p-value>0.05). It suggested that the organic solvents such as methanol, ethylene glycol and glycerin had no inhibitory effect on the ability of CD2-MnP to decolorize Indigo Carmine and Methyl Green.

Based on above results, CD2-MnP had a strong capability for decolorizing some dyes such as RBV5R, RBBR, Indigo Carmine and Methyl Green with the coexistence of organic solvents.

### Decolorization of simulated textile wastewater by the purified CD2-MnP

Considering the high concentration of salts and high ionic strength in textile effluents, the purified CD2-MnP was further evaluated for the decolorization of simulated textile wastewater containing different dye (details are described in Materials and methods). As shown in Table 4, purified CD2-MnP could effectively decolorize different simulated textile wastewater. The simulated textile wastewater containing Remazol Brilliant Violet 5R (10%, 30%), simulated textile wastewater containing Direct Red 5B (10%, 30%), simulated textile wastewater containing Remazol Brilliant Blue R (10%, 30%) and simulated textile wastewater containing Indigo Carmine (10%, 30%) could be decolorized up to 90.1%, 94.9%, 91.8%, 77.0%, 70.0%, 40.1%, 69.0%, 80.6% within 72 h by CD2-MnP, respectively (Table 4). The maximum decolorization of various simulated textile wastewater decreased with the increase of the initial concentration of simulated textile wastewater.

**Table 4:** Decolorization of simulated textile wastewater (10%, 30%, 50%) by purified CD2-MnP for 72 h.

Stimulated textile wastewater	Concentration(v/v)	Decolorization(%) after 72 h
RBV5R	10%	90.06
	30%	94.95
	50%	25.03
DR5B	10%	91.82
	30%	76.66
	50%	42.15
RBBR	10%	69.19
	30%	40.15
	50%	15.60
IC	10%	68.92
	30%	80.58
	50%	88.36

RBV5R: Remazol Brilliant Violet 5R; DR5B: Direct Red 5B; RBBR: Remazol Brilliant Blue R; IC: Indigo Carmine.

## Discussion

There existed some differences between the properties of CD2-MnP from *Irpex lacteus* CD2 and that of MnP from other organisms. For example, the optimal temperature of CD2-MnP was determined to be 70°C (Fig.2C), which was higher than MnP from *Phanerochaete chrysosporium* BKMF-1767 (30°C) [15], MnP from *Lentinula edodes* (40°C) [16] and MnP from *Schizophyllum* sp.F17 (35°C) [7]. Especially, UV-visible absorbance spectra of CD2-MnP suggested that this MnP was different from MnP of other organism. The absorption spectrum of CD2-MnP from *Irpex lacteus* CD2 showed maxima at 419 nm, 529 nm and 556 nm, which suggested that CD2-MnP was a heme protein with iron protoporphyrin IX as compound II. However, Shin et al. reported that the absorption spectrum of another MnP from *Irpex lacteus* strain KR 35W showed maxima at 407, 500, and 640 nm [30]. It indicated that MnP from *Irpex lacteus* strain KR 35 W was compound I by spectroscopical characterization [2], [29].

As shown in Fig.2D, there was evidence that the enzyme activity reduced slowly with time even at lower temperatures (such as 40°C). One reason for this phenomenon was that there still existed protein denaturation even at lower temperature [15], [31]. Previous research has reported that MnP from *Phanerochaete chrysosporium* was inactivated rapidly at temperature above 40°C [15]. Previous research also indicated that MnP was more susceptible to denaturation by temperature than LiP [31]. Another possible reason was the biphasic first-order model proposed by Liing and Lund [32] based on the presence of two groups with distinct thermal stabilities-a heat labile fraction that inactivates rapidly and a heat resistant fraction which cannot be inactivated completely [32], [33]. Thus we assumed that the heat-labile fraction of CD2-MnP may not tolerate 40°C. The enzyme activity reduced slowly with time even at lower temperatures (40°C).

In this study, it was found that the MnP activity of CD2-MnP was significantly inhibited by high concentration of Cd^2+^ (40 mM). Cd^2+^ in general was the inhibitor of enzymes. The Mn binding site of MnP was more flexible and allowed a broad range of metal ions to bind to its active site [34], [35]. Previous research about the kinetic analysis of the effect of cadmium on the activity of manganese peroxidase suggested that Cd^2+^ could bind to the Mn^2+^-binding sites, which prevented the oxidation of Mn^2+^ [36]. Therefore, Cd^2+^ was considered as a strong inhibitor of MnP. The possible reason for better tolerance to Mg and Ca than other ions was described as follows. Calcium was a component of binding sites of manganese peroxidase, and it has also been reported that calcium could maintain the structural stability of peroxidases [37]. Mg^2+^ was a cofactor of the enzyme peroxidase. Previous research also suggested that the binding of Mg^2+^ may stabilize and activate the manganese peroxidase [38]. In this study, it was found that the MnP activity of of CD2-MnP decreased when the concentration of organic solvents was increased to 30% (Fig.4B). It has been reported that the organic solvent had an inhibitory effect on the enzyme stability, because the organic solvent could affect the hydration shell of the enzyme molecule which was necessary for maintaining the native conformation [39]. Therefore, the inhibition of the activity of CD2-MnP by high concentration of organic solvents may be caused by the deformation of enzyme structure due to the hydrophobic effects.

Our results suggested that Ca^2+^, Mg^2+^ and Zn^2+^ could stimulate the MnP activity of CD2-MnP, which agreed with the previous research about the effect of metal ions on the activity of MnP purified from *Stereum ostrea* [14], *Rhizoctonia* [40] and *Schizophyllum* [7]. Although there have been some reports about the effect of different metal ions on the activity of MnP, few study has been performed to evaluate the influence of metal ions on the stability of MnP. Our research suggested that CD2-MnP from *Irpex lacteus* CD2 showed high stability in different metal ions (Table 2). To our knowledge, this is the first report about the effect of different metal ions on the stability of MnP. This character may be very valuable for the application of CD2-MnP in the treatment of wastewaters containing different metal ions.

Most previous research focused on studying the enzymatic and kinetic properties of MnP from different sources. However, the effect of different organic solvents on the MnP activity was rarely studied. Boer et al. have reported that the MnP purified from *Lentinula edodes* showed a high percentage of activity in reaction mixtures containing 10% (v/v) of different organic solvents such as acetone, isopropanol and ethanol. But the effect of higher concentration of organic solvents on the MnP activity was not investigated. The stability of MnP in different organic solvents was also not reported [16]. Our results showed that higher concentration of organic solvents such as glycerin, DMSO, ethylene glycol, isopropyl alcohol and butanediol (30%, v/v) had no inhibitory effect on the activity of CD2-MnP (Fig.3B). More importantly, our research indicated that CD2-MnP showed strong tolerance to many organic solvents especially ethanol, DMSO, butanediol and glycerin (Table 3). Thus, compared with the MnP from *Lentinula edodes* [16], CD2-MnP purified from *Irpex lacteus* CD2 in this study appeared to be more resistant to different organic solvents.

Previous research suggested that some azo dyes and anthraquinone dyes could be decolorized by the purified MnP from different fungal strains such as *Dichomitus squalens* [8], *Schizophyllum* [7], *Stereum ostrea* [14] and *Bjerkandera adusta* [41]. But as far as we know, no study has been performed to evaluate the decolorization capability of MnP in the presence of different metal ions or organic solvents. In this study, we found that the purified CD2-MnP from *Irpex lacteus* CD2 had a strong ability to decolorize different dyes with the coexistence of different metal ions or organic solvents (Fig.4 and Fig.6). This important property may contribute to the efficient use of MnP in the treatment of dye effluents.

## Conclusions

In this study, we found that the synthesis of manganese peroxidase in the white-rot fungus *Irpex lacteus* CD2 could be significantly enhanced by oxalic acid, veratryl alcohol and 2,6-Dimehoxyphenol. A novel manganese peroxidase named as CD2-MnP was purified and characterized from this fungus. CD2-MnP exhibited strong tolerance to different metal ions and organic solvents. The different types of dyes including the azo dye (Remazol Brilliant Violet 5R, Direct Red 5B), anthraquinone dye (Remazol Brilliant Blue R), indigo dye (Indigo Carmine) and triphenylmethane dye (Methyl Green) as well as simulated textile wastewater could be efficiently decolorized by CD2-MnP. CD2-MnP also had a strong capability for decolorizing different dyes with the coexistence of metal ions and organic solvents. In summary, the manganese peroxidase CD2-MnP from *Irpex lacteus* CD2 showed a great potential for the enzymatic degradation of different industrial dyes and textile dye effluents.

## Supporting Information

Figure S1 **A schematic representation of the enzyme reaction of manganese peroxidase (modified by reference [42]** **).**
(TIF)

Figure S2 **Induction of production of manganese peroxidase by various inducers (100 mg/L).**
(TIF)

Figure S3 **Comparison of the relative MnP activities of CD2-MnP at different metal ions (final concentration: 40 mM).** CK: without adding any metal compound; **highly significant difference, p-value<0.01.
(TIF)

Figure S4 **Comparison of the decolorization of dyes with the coexistence of different metal ions.** CK: without adding any metal compound; *significant difference, p-value<0.05; **highly significant difference, p-value<0.01. RBV5R: Remazol Brilliant Violet 5R, DR5B: Direct Red 5B, RBBR: Remazol Brilliant Blue R, IC: Indigo Carmine, MG: Methyl Green.
(TIF)

Figure S5 **Comparison of the decolorization of dyes with the coexistence of different organic solvents.** CK: without adding any organic solvent; **highly significant difference, p-value<0.01. RBV5R: Remazol Brilliant Violet 5R, DR5B: Direct Red 5B, RBBR: Remazol Brilliant Blue R, IC: Indigo Carmine, MG: Methyl Green.
(TIF)
