# Supplementary figures and images for: Induction, Purification and Characterization of a Novel Manganese Peroxidase from Irpex lacteus CD2 and Its Application in the Decolorization of Different Types of Dye
Source: PLoS One. 2014 Nov 20;9(11):e113282. doi: 10.1371/journal.pone.0113282 (PMC4239052; doi:10.1371/journal.pone.0113282)

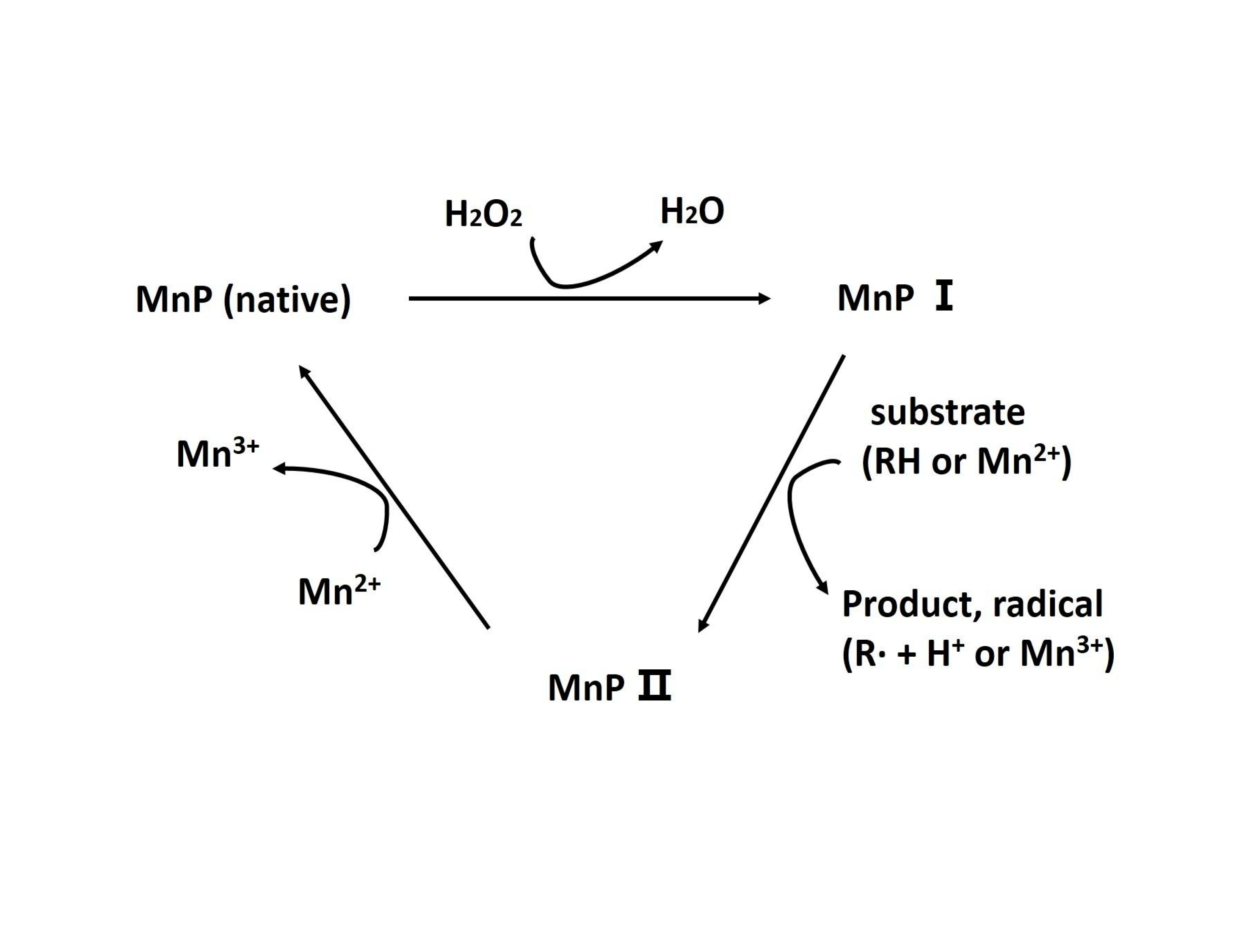

Supplement: Figure S1 — A schematic representation of the enzyme reaction of manganese peroxidase (modified by reference [42] ). (TIF) [file pone.0113282.s001.tif]

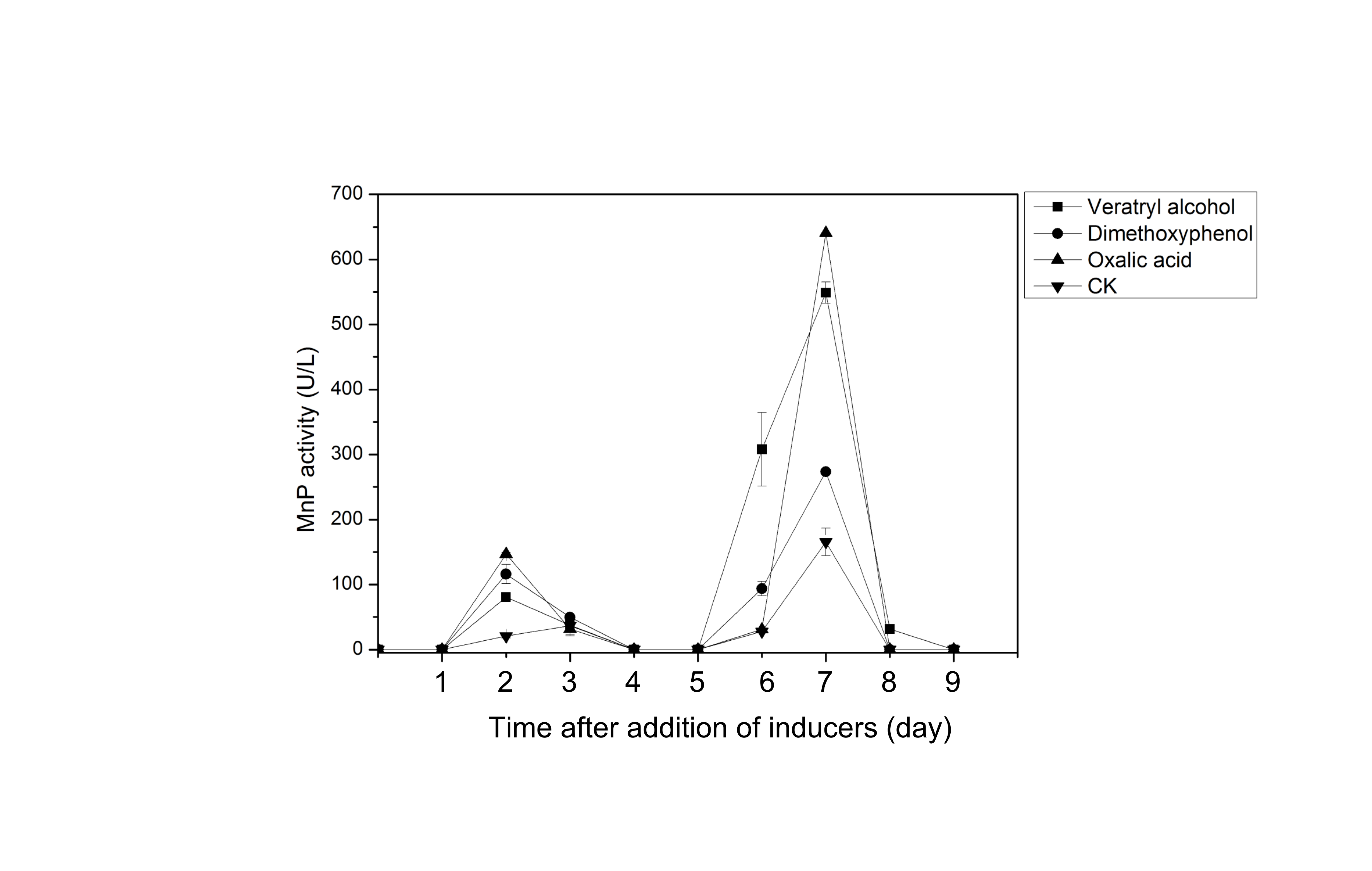

Supplement: Figure S2 — Induction of production of manganese peroxidase by various inducers (100 mg/L). (TIF) [file pone.0113282.s002.tif]

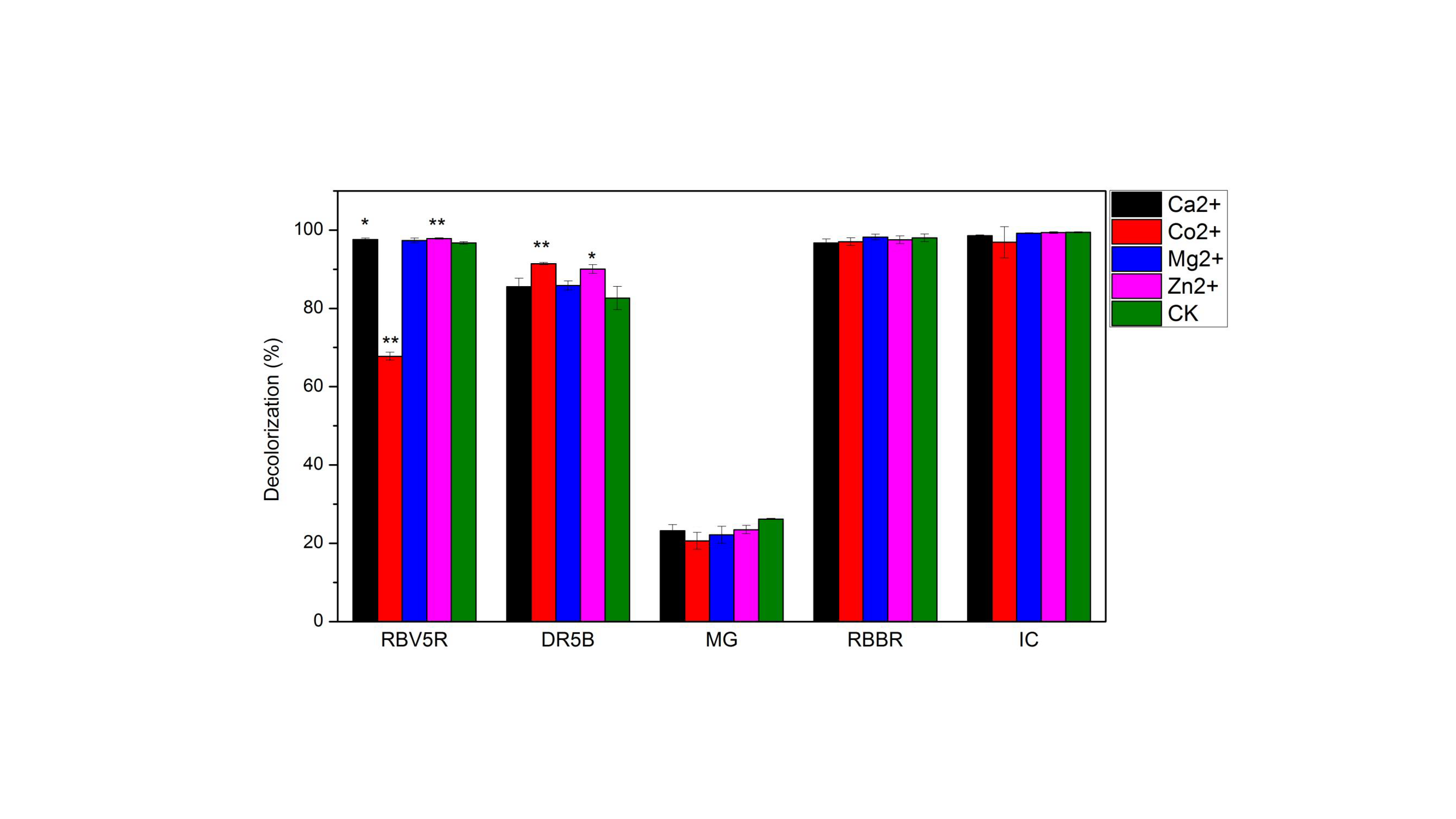

Supplement: Figure S4 — Comparison of the decolorization of dyes with the coexistence of different metal ions. CK: without adding any metal compound; *significant difference, p-value<0.05; **highly significant difference, p-value<0.01. RBV5R: Remazol Brilliant Violet 5R, DR5B: Direct Red 5B, RBBR: Remazol Brilliant Blue R, IC: Indigo Carmine, MG: Methyl Green. (TIF) [file pone.0113282.s004.tif]

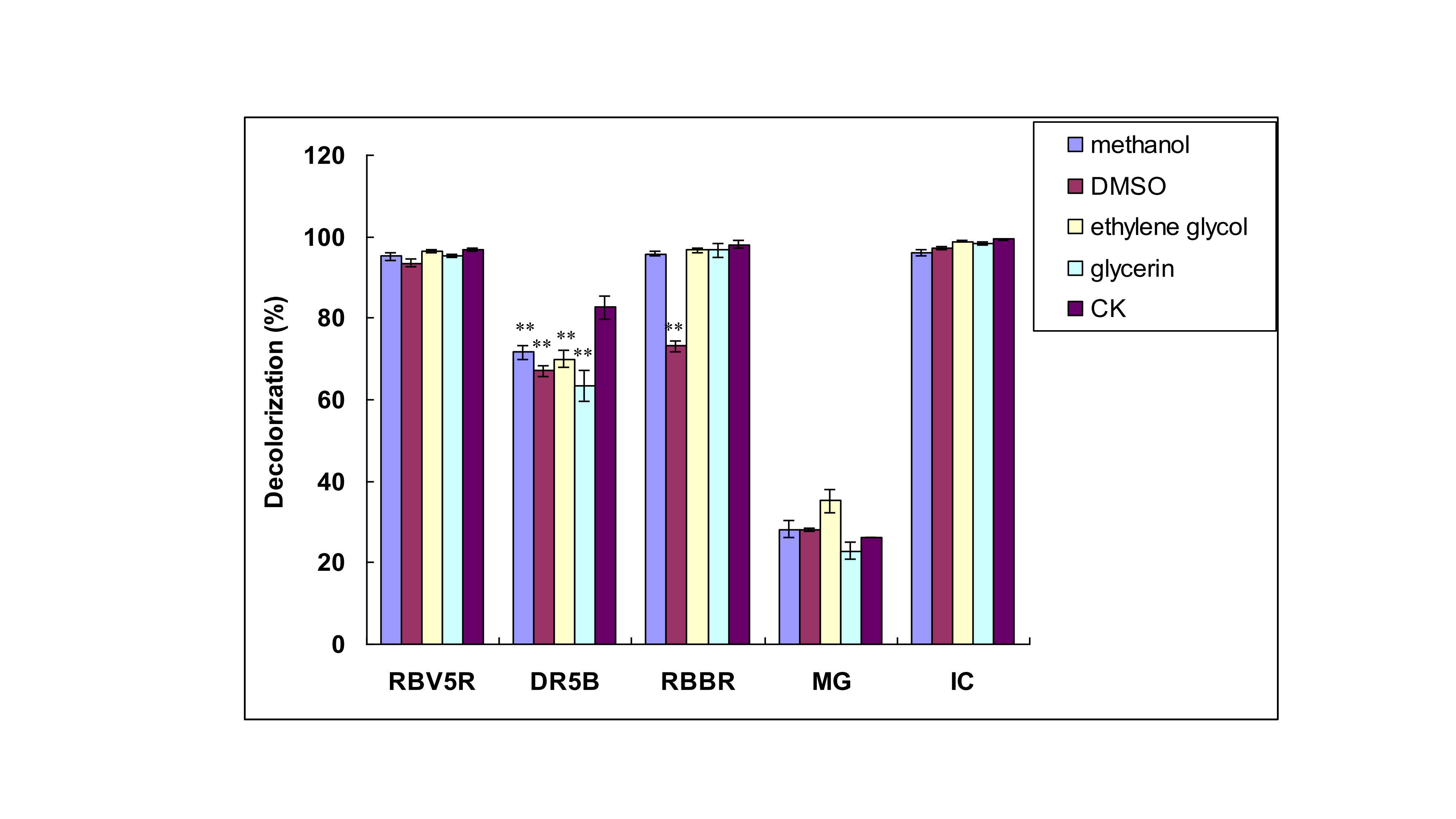

Supplement: Figure S5 — Comparison of the decolorization of dyes with the coexistence of different organic solvents. CK: without adding any organic solvent; **highly significant difference, p-value<0.01. RBV5R: Remazol Brilliant Violet 5R, DR5B: Direct Red 5B, RBBR: Remazol Brilliant Blue R, IC: Indigo Carmine, MG: Methyl Green. (TIF) [file pone.0113282.s005.tif]
